# The Role of Quality Health Services and Discussion about Birth Spacing in Postpartum Contraceptive Use in Sindh, Pakistan: A Multilevel Analysis

**DOI:** 10.1371/journal.pone.0139628

**Published:** 2015-10-20

**Authors:** Hannah Tappis, Anis Kazi, Waqas Hameed, Zaib Dahar, Anayat Ali, Sohail Agha

**Affiliations:** 1 Jhpiego/USA—an affiliate of Johns Hopkins University, Baltimore, Maryland, United States of America; 2 Heartfile, Islamabad, Pakistan; 3 Marie Stopes Society, Karachi, Pakistan; 4 Jhpiego/Pakistan, Karachi, Pakistan; NHS lothian and University of Edinburgh, UNITED KINGDOM

## Abstract

**Introduction:**

Rapid population growth, stagnant contraceptive prevalence, and high unmet need for family planning present significant challenges for meeting Pakistan’s national and international development goals. Although health behaviors are shaped by multiple social and environmental factors, research on contraceptive uptake in Pakistan has focused on individual and household determinants, and little attention has been given to community characteristics that may affect access to services and reproductive behavior.

**Methods:**

Individual and community determinants of contraceptive use were identified using multivariable multilevel logistic regression to analyze data from a 2014 cross-sectional survey of 6,200 mothers in 503 communities in Sindh, Pakistan.

**Results:**

Only 27% of women who had given birth in the two years before the study reported using contraceptives. After adjusting for individual and community characteristics, there was no difference in the odds of contraceptive use between urban and rural women. Women who had delivered at a health facility had 1.4 times higher odds of contraceptive use than women who delivered at home. Those who received information about birth spacing from a doctor or relatives/friends had 1.81 and 1.38 times higher odds of contraceptive use, respectively, than those who did not. Living in a community where a higher proportion of women received quality antenatal care and where discussion of birth spacing was more common was significantly associated with contraceptive use. Community-wide poverty lowered contraceptive use.

**Conclusions:**

Quality of care at the community level has strong effects on contraceptive use, independent of the characteristics of individual households or women. These findings suggest that powerful gains in contraceptive use may be realized by improving the quality of antenatal care in Pakistan. Community health workers should focus on generating discussion of birth spacing in the community. Outreach efforts should target communities where the demand for contraception appears to be depressed due to high levels of poverty.

## Introduction

Family planning has the potential to reduce maternal and child mortality and contribute to poverty reduction and social and economic development, particularly in countries with high fertility and rapid population growth [1–3]. Although family planning is the most cost-effective and practical strategy for reducing preventable maternal and newborn mortality, there are more than 220 million women in the developing world who want to avoid or postpone childbearing but are not using any form of modern contraception [4].

The implications of low contraceptive prevalence are especially striking in Pakistan, the sixth most populous country in the world, which has a higher fertility rate (3.8 births per woman) and a faster population growth rate (2% per year) than many of its South Asian and eastern Mediterranean neighbors [5, 6]. The current contraceptive prevalence rate among women ages 15–49 is 35% (23% in rural areas); unmet need among married women is 20%; and the median interval between births is 28 months [6]. Although the World Health Organization recommends an interval of at least 24 months between childbirth and the next pregnancy to reduce the risk of adverse maternal, perinatal, and infant outcomes, more than one-third of Pakistani children are born less than 24 months after a previous birth [6, 7]. Without dramatic increases in family planning and consequent fertility decline, the country’s population is projected to grow from an estimated 184.5 million in 2012–2013 to between 266 million and 342 million by 2050 [5, 8].

At the 2012 London Summit on Family Planning, Pakistan committed to increasing contraceptive prevalence to 55% by 2020 [9]. The country’s commitment to family planning is not new; however, the increases in contraceptive use that have been attributed to the establishment of the government programs and social franchising clinics launched in the 1990s have slowed in recent years [10–12]. For example, contraceptive prevalence increased significantly after introduction of the National Program for Family Planning and Primary Health Care, which established a cadre of community-based Lady Health Workers in 1994, but then stagnated due to suboptimal counseling sessions, supply management issues, and diversion of health workers’ attention to promotion of other health services. [12, 13] There has been little change in contraceptive use since 2000 [14, 15]. Similarly, the initial progress after the launch of social marketing media campaigns and establishment of franchised family planning clinics in urban areas has not been sustained [16, 17].

Today, family planning services are available at low or no cost from multiple public and private sector outlets in Sindh. This includes hospital-based Reproductive Health Service Centers that provide comprehensive family planning services in urban areas; Family Welfare Centers that provide all services except for contraceptive implants, tubal ligataion and vasectomy in urban and rural areas; Mobile Service Units that set up ‘camps’ to provide services in underserved rural areas, community-based Social Mobilizers (Male) that distribute condoms and oral contraceptive pills; and Lady Health Workers that provide counseling on family planning as well as condoms, oral contraceptive pills and injectables; private facilities; and social marketing organizations.[18]

Poor-quality service provision has been a major barrier to scaling up use of contraception. Giving information to clients about the benefits and potential side effects of modern methods of contraception is not sufficient [19]. For example, although 74% of couples using contraceptives do so to limit the number of children they have, only 30% select long-acting or permanent contraceptive methods. Furthermore, 72% of users are not informed about the potential side effects of contraceptives and how to address them, leading to high rates of discontinuation [6]. As in other settings, the concerns that women in Pakistan raise about service quality include providers who lack knowledge of contraception, low availability of a variety of contraceptive methods, and provider unresponsiveness to women’s queries and apprehensions about family planning [20].

Two additional barriers to contraceptive uptake are women’s perception that family planning is socially or culturally unacceptable and their perception that contraceptive use could conflict with their husband’s or mother-in-law’s fertility preferences [21]. Women’s intention to use contraception is hindered by their beliefs that family planning decisions must be made by the husband and that fertility is determined by God's will. For men, the fear that contraceptives make a woman sterile and may harm her womb is a barrier to using modern family planning methods [22]. In this context, couples’ joint decision-making has been shown to have a significant effect on contraceptive use [23].

Health behaviors are shaped by multiple social and environmental factors, and the influence of these factors on individual beliefs and behaviors has been given increased attention by social scientists in recent years [22–25]. Both the access to resources and the influence of family, friends, and broader social norms affect access to health providers, health knowledge, and contraceptives [24]. However, there is little research on Pakistan showing how the characteristics of the community where a woman lives influence her contraceptive use. Most research has focused on individual socioeconomic factors, spousal communication about family planning, and the influence of the mother-in-law [19, 21–23, 25–28].

Research in other settings has shown that women living in communities with relatively high quality health services and women in communities with relatively low poverty rates are more likely to seek reproductive care services. [29–33] Given that use of family planning continues to carry stigma in the conservative cultural environment of Pakistan, there is also a need to look at communities’ openness to discussing family planning as a factor that could have a positive influence on contraceptive use [22]. Community openness to discussing prevention has been a major contributing factor in health behavior change, and both interpersonal communication and peer support are recognized as key components of an enabling environment for family planning programming [34, 35]. This study examines the individual and community-level determinants of contraceptive use among women within two years of giving birth in Sindh, Pakistan’s second most populous province, with the aim of understanding how family planning decisions are influenced by the characteristics of the communities in which they live.

## Methods

This study uses data from a cross-sectional survey conducted in June–July 2014 as part of a United States Agency for International Development-funded program to improve maternal, newborn, and child health outcomes by supporting the delivery of evidence-based, high-impact interventions in the Sindh province of Pakistan.

### Study sample

A multistage sampling procedure was used to select 6,200 women ages 15–49 who had a live birth in the last two years. The sample, drawn from the sampling frame based on the 1998 census (the most recent census conducted in Pakistan), was selected to be representative of all districts of Sindh.

Probability proportional to size sampling was used to select urban areas in each district. Urban areas were divided into three strata based on population size: cities of more than 1 million, cities of 0.1–1 million, and cities of less than 0.1 million. In each district, cities were listed in descending order of size and selected using a skip interval (determined by dividing the cumulative population of the cities by the number of cities to be selected in the sample) after the first city was selected at random using a random number table. This process of city selection was applied within each stratum of each district. Within urban areas, “circles” were the primary sampling units (PSUs), and they were also selected using probability proportional to size sampling. Within a circle, a random starting point was used to select the first household.

In rural areas, villages served as the PSUs. Villages were stratified according to population size (greater than or less than 3,000). In each stratum, villages were listed in descending order based on population size. Villages in a stratum were selected by using a skip interval (obtained by dividing the total population of villages in that stratum by the number of villages to be selected). Villages were divided into two parts, and random starting points were used to select the first households in each part.

Finally, in both urban and rural areas, if more than one eligible individual was found in a household, a Kish grid was used to select one respondent randomly.[36] If the selected respondent was not present, two attempts were made to reach the respondent after the initial contact If still not available after a total of three attempts, a replacement was selected. In total, 7458 households were selected to reach 6200 respondents (16.9% non-response). Smaller districts were oversampled for better representation of smaller areas, and weights were attached to the data to adjust for oversampling. Urban and rural populations are proportionately represented in each district.

### Data collection and management

Data were collected through face-to-face interviews using a structured questionnaire based on the Demographic and Health Survey instrument developed by Macro International, Inc., and the Knowledge, Practice and Coverage Survey developed by the Johns Hopkins University/Child Survival Program 1990 and revised by the Maternal and Child Health Integrated Program [6, 37]. Questionnaires were translated into Urdu and Sindhi, and data were collected on paper forms by experienced female data collectors. Data entry and cleaning were done by s reputable research firm contracted to manage the fieldwork and data entry for this study.

### Measures

#### Outcome variable

The outcome variable of interest in this study is current contraceptive use. Women were asked if they were currently doing something to delay or avoid getting pregnant, and responses were coded as a binary variable: 0 if the woman was not doing anything to delay or avoid getting pregnant, and 1 if the woman was using any modern (pill, injection, intrauterine device, implant, male or female sterilization, standard days method, or lactation amenorrhea method) or traditional (rhythm, withdrawal, or other) method.

#### Independent variables

The individual and household (level 1 variables) captured included each woman’s place of residence (urban/rural), age (continuous), number of children (one, two, or three or more), level of school attended (no formal education, primary or middle school, secondary or higher education), current desire for more children (yes/no), and daily television viewership (yes/no). In addition, place of delivery for the woman’s most recent live birth (home/facility) was recorded, as was whether she received information about birth spacing from any of the following four individuals in the last 12 months (four separate variables): a doctor, a Lady Health Worker, her mother-in-law, or any other relative/friend. A variable measuring household wealth was created with the method used by the Demographic Health Surveys, using principal component analysis, with data collected on the following assets and amenities: ownership of mobile phone, motorcycle, television, refrigerator, cupboard/cabinet, washing machine, bed, clock, sofa, sewing machine, livestock; construction material used for the floor of the home; construction material used for the roof of the home; construction material used for the walls of the home; main fuel used by the household; whether the household had a water pump; and whether the household had a toilet [37].

Three community (level 2) variables were constructed by aggregating individual-level data for each of the 503 communities/clusters/PSUs: one to indicate availability of quality antenatal care, one to indicate openness to communication about family planning, and one to indicate poverty status. The variable measuring quality of antenatal care in the PSU was constructed in two steps: individual women were coded with a 1 if they had received all six elements of quality antenatal care (blood pressure measurement, urine test, blood test, iron tablets, tetanus immunizations, and weight measurement) during an antenatal care visit and with a 0 if they had not; in the second step, the mean value of this variable was calculated for the PSU. Similarly, the variable measuring discussion of family planning in the community was constructed in two steps: the first variable measured whether a woman received information on birth spacing by any of four persons (doctor, Lady Health Worker, mother-in-law, or any other relative or friend); thereafter, the mean value of this variable was calculated for the PSU. Finally, community-level poverty was computed in a similar manner: at the individual level, women belonging to the poorest wealth quintile were coded with a 1 and women in all other quintiles were coded with a 0; then, the average value was calculated for the PSU. This approach is similar to that of previous studies in which individual-level data have been used to create community-level variables by calculating the means of individual-level variables at the PSU level [33, 38, 39].

### Statistical analysis

Descriptive, bivariate, and multivariate logistic regression techniques were used to analyze survey findings.

First, individual and household characteristics of respondents were explored using descriptive statistics and contraceptive prevalence depicted in spatial maps. Bivariate analyses were then conducted using Pearson’s chi-square to examine relationships between study sample characteristics and contraceptive use. Because respondents living in the same community are likely to be more similar to each other than to respondents in other PSUs, and some determinants of contraceptive use may be a function of conditions in that community, two-level logistic regression models were developed with the following structure:

$$\gamma_{ij}=\pi_{ij}+e_{ij}$$ (1)

where *π*_ij_ is the probability of the *i*th woman in the *j*th cluster/PSU using contraception. The response variable, *y*_ij_, is distributed as binomial (1, *π*_ij_). *e*_ij_ is the residual term at level 1, a random variable, with variance of *π*_ij_(1- *π*_ij_).

$$\text{log}\left(\pi_{ij}/1-\pi_{ij}\right)=\alpha +\beta {\text{Χ}}_{\pi_{ij}}^{\text{Τ}}+U_{j}$$ (2)

The outcome fitted in the model is the log odds of use versus non-use of contraception. α is a constant and β is a vector of independent variables. U_j_ is a random intercept for community, defined as U_j_ ~ N(0,σ_u_^2^).

#### Random effects, model fit, and precision

Correlations between the probability of contraceptive use in the same PSU were computed using an inter-cluster correlation/variance partition coefficient (VPC_f = σ_^2^_f_ / (_(σ_^2^_f_ +3.29)), where _σ_^2^_f_ is a measure of variance at the community level.

A block modeling strategy was used, whereby a set of variables is included in a model separately, resulting three different models: (1) a null model, to decompose the total variance between individual and community levels; (2) a second model, containing all individual-level variables; and (3) a final model, incorporating all three community-level variables to assess their effect and examine whether the association between individual-level variables and contraceptive use remained significant. Women’s age was dropped from the regression analyses due to the issue of multicollinearity as it was highly correlated with number of living children. The final model, incorporating individual and community-level factors, was selected as the best fit based on the likelihood ratio test, Akaike information criterion, Bayesian information criterion, and two-tailed Wald statistics.

Stata version 11.2 (StataCorp LP, Texas, United States) was used for the analyses, and *p*-values of 0.05 were considered statistically significant. ARCGIS 10.2.1 (ESRI, California, United States) was used for spatial analysis.

### Ethical considerations

Study participants included married women 15–49 who had had a live birth in the two years prior to the survey and who resided in the houses sampled for study participation. All individuals meeting these criteria were considered adults, regardless of age. Interviewers obtained oral consent from each prospective participant prior to conducting an interview. An informed consent script was read aloud to the prospective participants, and she was asked if she had any questions. All respondents’ questions were answered before participants were asked to provide oral consent. Interviewers were instructed to reassure women that they were not obligated to take part in the study if they did not want to and could stop the interview at any time. After obtaining verbal consent from the respondent, the interviewer signed the consent form, put the questionnaire number on it, and kept it in the study records. Written informed consent was not taken because of low female literacy in the study area. All consent procedures were approved by the by both the Johns Hopkins University School of Public Health Institutional Review Board and the National Bioethics Committee of Pakistan

## Results

### Study population characteristics

Table 1 presents the characteristics of women within two years postpartum in Sindh, of whom 69.1% delivered in a health facility. Nearly equal proportions were living in rural (51.2%) and urban (48.8%) localities. The median age of the women was 27 and the median number of living children was three. Approximately two-thirds (67.6%) of the women expressed desire for another child in the future. More than half (55.5%) had never attended school; 20.9% had completed primary or middle school; and 23.6% had a secondary or higher education. Approximately two out of five women (43.0%) reported watching television daily. Respondents were asked if they had received information regarding birth spacing during the previous 12 months. More than one-third (39.3%) had received information from a doctor, 35.2% had received information from relatives or friends, 22.9% had received information from a Lady Health Worker, and 18.7% had received information from their mother-in-law.

**Table 1:** Characteristics of women within two years postpartum in Sindh, Pakistan, 2014.

Characteristics	% (n = 6,200)
**Sociodemographic characteristics**	
Place of residence	
Rural	51.2
Urban	48.8
Number of living children	
One	22.6
Two	23.1
Three or more	54.2
Desire for additional children	
Want additional children	67.6
Do not want additional children	32.4
Education	
None	55.5
Primary or middle	20.9
Secondary or higher	23.6
Daily television viewership	
Do not view television daily	57.0
View television daily	43.0
**Health services use**	
Place of delivery for most recent birth	
Home	39.0
Facility	69.1
**Discussion about birth spacing**	
Discussed birth spacing with doctor in last 12 months	
No	60.7
Yes	39.3
Discussed birth spacing with mother-in-law in last 12 months	
No	81.3
Yes	18.7
Discussed birth spacing with relatives or friends in last 12 months	
No	64.8
Yes	35.2
Discussed birth spacing with Lady Health Worker in last 12 months	
No	77.1
Yes	22.9

### Contraceptive use

**Fig 1:**
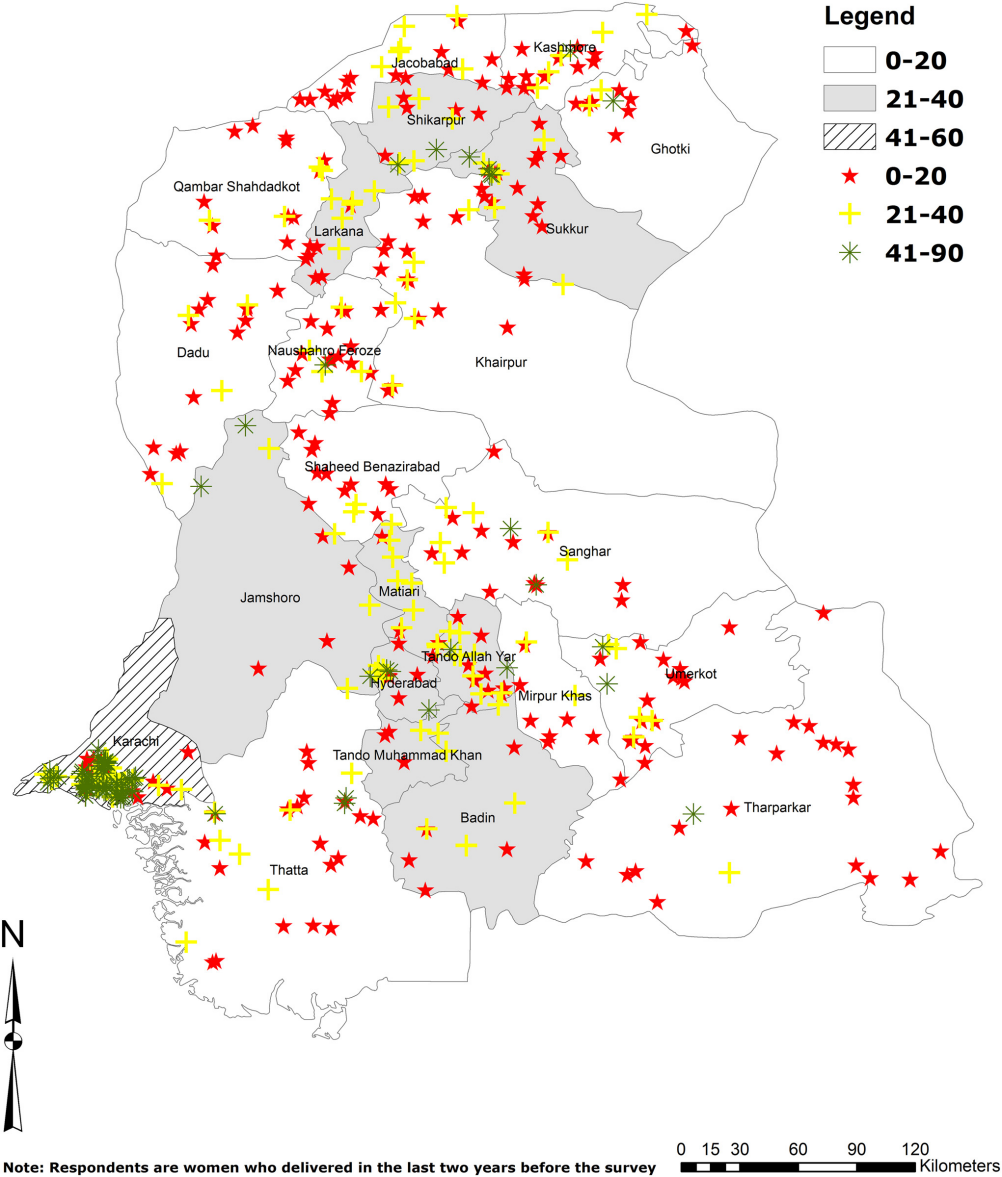
Map of average contraceptive prevalence by community and district in Sindh, Pakistan, 2014. Shaded areas represent the average contraceptive prevalence rate at the district level. Colored symbols represent the average contraceptive prevalence rate at the community level.

**Fig 2:**
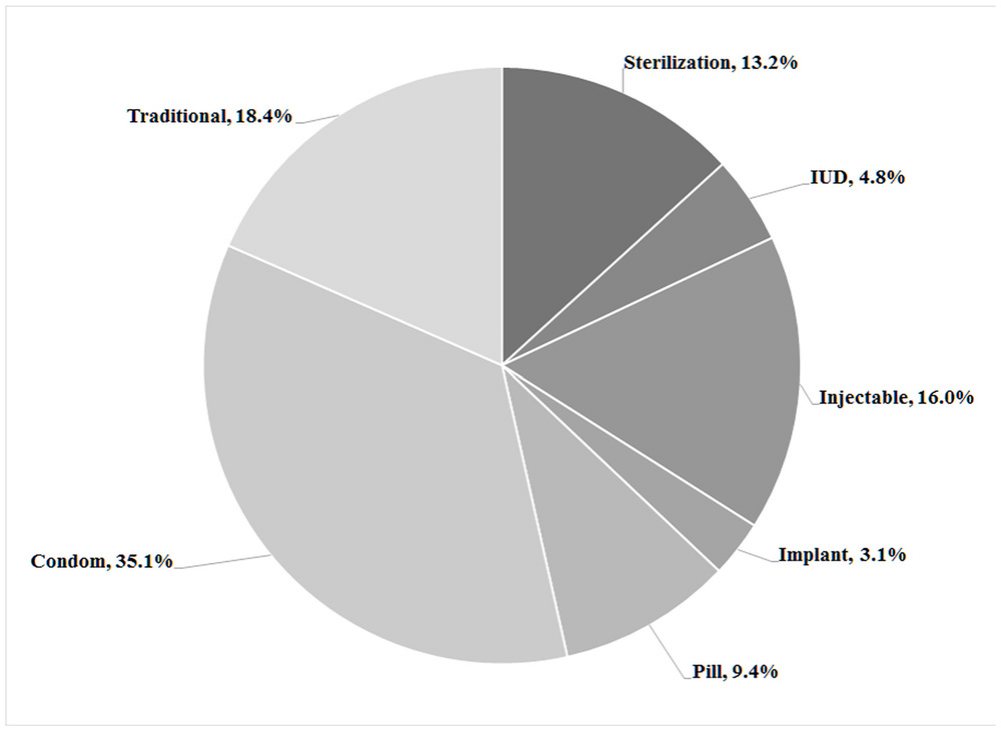
Method mix among women within two years postpartum using any form of contraception, Sindh, Pakistan, 2014.

Overall, 27.3% of women with a live birth in the two years before the study reported contraceptive use. Fig 1 illustrates the average prevalence of contraceptive use in each community (PSU) and district, and Fig 2 presents the contraceptive methods used. Although in Karachi, which accounts for approximately one-third of the province’s population, 45.2% (95% CI: 41.6, 48.9) of women within two years postpartum reported contraceptive use at the time of the survey, in 14 of the 23 other districts 21%–40% of respondents reported contraceptive use, and in nine districts less than 20% of the women reported contraceptive use. Approximately one-fifth (18.4%) of postpartum contraceptive users practiced traditional methods, while fourth-fifths (81.6%) used modern methods.

Table 2 presents the percentage of women reporting use of contraceptives at the time of the survey, by sociodemographic characteristics, health service use, and exposure to communication about birth spacing. Nearly two-fifths (39.3%) of women living in urban areas reported using contraceptives, compared to only 16% in rural areas. Contraceptive use was reported by more than twice as many women who delivered their most recent child at a health facility (32.7%) as those who did not deliver at a facility (15.3%). Contraceptive use was also more common among women with higher wealth status, those with more education, and those who reported having received information about birth spacing from a health worker, family member (other than mother-in-law), or friend in the last 12 months.

**Table 2:** Percentage of women within two years postpartum using any contraceptive method at the time of the survey, Sindh, Pakistan, 2014.

Characteristics	% (n = 6,200)
**Sociodemographic characteristics**	
Place of residence	
Rural	16.0***
Urban	39.3
Number of living children	
One	17.0***
Two	29.5
Three or more	30.8
Desire for additional children	
Want additional children	63.0***
Do not want additional children	37.0
Education	
None	18.3***
Primary or middle	31.8
Secondary or higher	44.6
Household Wealth Quintiles	
First	8.5***
Second	17.3
Middle	29.0
Fourth	35.6
Richest/Fifth	46.6
Daily television viewership	
Do not view television daily	20.4***
View television daily	36.6
**Health services use**	
Place of delivery for most recent birth	
Home	15.3***
Facility	32.7
**Discussion about birth spacing**	
Discussed birth spacing with doctor in last 12 months	
No	20.0***
Yes	38.6
Discussed birth spacing with mother-in-law in last 12 months	
No	26.6**
Yes	30.7
Discussed birth spacing with relatives or friends in last 12 months	
No	23.0***
Yes	35.4
Discussed birth spacing with Lady Health Worker in last 12 months	
No	25.6***
Yes	33.4

*significant at *p*< 0.05

**significant at *p*< 0.01

***significant at *p*< 0.001

### Determinants of contraceptive use

Multilevel logistic regression analysis was conducted to determine the effect of individual and community-level variables on current use of contraception. The results are shown in Table 3. The null model shows significant differences in contraceptive use across communities/PSUs (community-level variance = 0.95; SE = 0.12), and VPC indicates that 22% of the variation in women’s propensity to use contraception lies between communities/PSUs.

**Table 3:** Associations between individual/household and community-level factors and contraceptive use in Sindh, Pakistan.

Characteristic	Null model (Model 1)	Model with individual/ household- factors only (Model 2) Adjusted odds ratio	Model with individual/household and community factors (Model 3) Adjusted odds ratio
**INDIVIDUAL & HOUSEHOLD CHARACTERISTICS**			
Urban residence (ref: rural)		1.40 (1.11, 1.77)	1.0 (0.77, 1.29)
Number of children (ref: one)			
Two		2.09 (1.68, 2.60)	2.09 (1.68, 2.60)
Three or more		3.14 (2.55, 3.86)	3.16 (2.57, 3.89)
Wants no more children (ref: want more children)		2.12 (1.83, 2.46)	2.09 (1.80, 2.42)
Education (ref: none)			
Primary or middle		1.21 (1.00, 1.45)	1.17 (0.97, 1.41)
Secondary or higher		1.96 (1.59, 2.41)	1.90 (1.54, 2.33)
Household wealth (ref: poorest quintile)			
Second quintile		1.67 (1.28, 2.18)	1.29 (0.97, 1.75)
Middle quintile		2.40 (1.79, 3.21)	1.68 (1.19, 2.38)
Fourth quintile		2.49 (1.79, 3.46)	1.70 (1.16, 2.49)
Richest/fifth quintile		3.50 (2.45, 4.99)	2.38 (1.59, 3.56)
Daily television viewership (ref: no)		1.21 (1.04, 1.41)	1.18 (1.01, 1.37)
**HEALTH SERVICES USE**			
Last birth at a health facility (ref: no)		1.46 (1.22, 1.73)	1.41 (1.18, 1.67)
**Discussion about birth spacing**			
Discussed birth spacing with doctor in last 12 months (ref: did not)		1.87 (1.62, 2.16)	1.81 (1.56, 2.09)
Discussed birth spacing with mother-in-law in last 12 months (ref: did not)		0.89 (0.74, 1.06)	0.89 (0.74, 1.06)
Discussed birth spacing with relative or friend last 12 months (ref: did not)		1.42 (1.22, 1.65)	1.38 (1.18, 1.60)
Discussed birth spacing with Lady Health Worker in last 12 months (ref: did not)		1.19 (1.01, 1.40)	1.15 (0.98, 1.36)
**COMMUNITY CHARACTERISTICS**			
Concentration of impoverished households			0.47 (0.28, 0.77)
Quality health services available			2.29 (1.41, 3.72)
Discussion of birth spacing occurs			1.50 (1.10, 2.23)
**RANDOM EFFECTS & MULTIVARIATE MODEL FIT**			
N (number of communities/number of women)	503 (6,200)	503 (6,200)	503 (6,200)
Community level variance (SE)	0.95 (0.12)	0.40 (0.07)	0.34 (0.07)
Community level VPC (%)	22.4%	10.8%	9.4%
Log likelihood	-3,339.098	-2,907.608	-2,893.359
Akaike information criterion	6,682.195	5,851.216	5,828.718
Bayesian information criterion	6,695.6	5,972.397	5,970.097

#### Individual and household-level factors

Model 3 shows that when controlling for other individual/household factors and both measured and unmeasured community-level factors, the odds of contraceptive use increases with the number of living children a woman has. Women with two children have an estimated 2.09 times greater odds of contraceptive use than those with one child (95% CI: 1.68, 2.60); and women with three or more children have 3.16 times greater odds of contraceptive use than those with one child (95% CI: 2.57, 3.89). The model predicts that, after controlling for other individual characteristics and both measured and unmeasured community-level factors, women who have completed secondary or higher education have 1.90 times greater odds of contraceptive use than women with no education(95% CI: 1.54, 2.33). Women who expressed a desire to limit fertility had 2.09 times greater odds of contraceptive use than those who wanted more children (95% CI: 1.80, 2.42). Wealth quintile had a substantial positive effect on the use of contraception: women in the richest quintile had 2.38 times greater odds of using contraception compared with those who belonged to the poorest group (95% CI: 1.59, 3.56). The odds of using contraception was 1.41 times higher among women who had their last delivery in a health facility than among women who delivered at home (95% CI: 1.18, 1.67). Daily television viewership also significantly increased the odds of contraception by a factor of 1.18 (95% CI: 1.01, 1.37).

Women who received information about birth spacing from a doctor and women who received the information from relatives/friends (in the previous 12 months) had 1.81 times (95% CI: 1.56, 2.09) and 1.38 times (95% CI: 1.18, 1.60) higher odds of contraceptive use, respectively, than those who did not receive information about birth spacing. There was not a significant difference in contraceptive uptake between women who received information about birth spacing from their mother-in-law and those who did not. Although receiving information about birth spacing from a Lady Health Worker was associated with contraceptive use, when controlling for other individual and household factors (Model 2), this effect ceased to be statistically significant when community-level factors were taken into account. In particular, the effect of receiving birth spacing information from a Lady Health Worker became non-significant when the variable measuring community-level discussion of birth spacing was taken into account (data not shown). Similarly, women living in urban localities had 1.4 times higher odds of contraceptive use than those living in rural areas (95% CI: 1.1, 1.8), when controlling for individual and household factors (Model 2). However, place of residence ceased to be statistically significant when community-level factors were taken into account (Model 3). In particular, it was the addition of the variable that measured community-level quality of antenatal care that reduced the statistical significance of the effect of urban residence on contraceptive use (data not shown).

#### Community-level factors

As the concentration of poor households in a community increased, the odds of contraceptive use for an individual woman in that community substantially declined (adjusted odds ratio, 0.47; 95% CI: 0.28, 0.77). Moreover, as the proportion of women in a community who received quality antenatal care increased, so did the odds of contraceptive use for a woman in that community (adjusted odds ratio, 2.29; 95% CI: 1.41, 3.77). Similarly, the more common it was for community members to have had discussions about birth spacing in the last 12 months, the more likely it was for a woman living in that community to report contraceptive use (adjusted odds ratio, 1.50; 95% CI: 1.10, 2.23).

## Discussion

This study considers a broader range of influences on contraceptive use than have been examined by previous studies of family planning determinants in Pakistan. Results show that community characteristics were significantly associated with an individual woman’s propensity for family planning. Specifically, living in a community where women receive quality antenatal care and where there is discussion of birth spacing had a positive effect on contraceptive use. Powerful gains in contraceptive use could be realized by improving the quality of antenatal care, including counseling on postpartum family planning, as well as by supporting community health workers to engage community members in discussions about healthy timing and spacing of pregnancy.

The findings also show that community-level economic disadvantage was associated with lower contraceptive use, even after individual socioeconomic disadvantage was taken into account. Focusing efforts to improve education and service provision in communities where the demand for contraception appears to be depressed due to high levels of poverty could also increase contraceptive uptake. The finding that the effect of urban residence disappeared after taking community-level variables—specifically, the quality of antenatal care at the community level—into account suggests that the urban/rural differential in contraceptive use in Pakistan is a function of urban communities having access to better-quality services.

The independent effect of living in a community where women receive quality antenatal care, coupled with the increased odds of contraceptive use associated with having given birth at a health facility and having received information about birth spacing from a doctor, suggest that interaction with maternal health services increases the likelihood of contraceptive use. Use of maternal health services increases familiarity with service providers and may strengthen relationships with health workers and/or trust in the health system, increasing the likelihood of using other reproductive health services in the future [11, 40]. Furthermore, the positive effect of living in a community where women receive quality antenatal care suggests that the content and process of care are also important to contraceptive uptake. This is important because there is little empirical evidence that directly links the quality of reproductive or maternal health services to contraceptive adoption. At present, the evidence that quality of care is a barrier to contraceptive use in Pakistan is largely limited to studies in which women explain reasons for not using contraceptives in terms of side effects of concerns about their health [41]. How the quality of services directly or indirectly affects contraceptive use has not been established. One explanation for the link between living in a community where women access quality antenatal care and contraceptive uptake is that health facilities that provide the six services that define quality antenatal care are more likely to provide counseling on postpartum family planning as part of routine maternal health services. Another is that facilities that provide quality antenatal care are more likely to have the human resources and supplies required to provide quality family planning services, so quality antenatal care serves as a proxy indicator for general quality of reproductive and maternal health services. Both interpretations are consistent with global consensus that building stronger primary health care systems and optimizing integration of family planning services may lead to greater contraceptive uptake [42–45].

The findings related to the discussion of birth spacing also have important implications for programs. The relationship between receiving family planning information from a doctor and contraceptive use may be another indicator of the importance of health service interactions, or may be a reflection of the power and influence doctors have in Pakistani society [46–48]. Although it is surprising that a woman’s discussion of birth spacing with her mother-in-law did not have a significant effect on her contraceptive use, it is possible that women’s perceptions of their mother-in-law’s opinion carry more weight than actual discussions of family planning, or that the effect observed in other studies was masked in our analysis by the influence of other relatives. Couples’ discussion of contraception also has been shown to lead to desired pregnancy outcomes in Pakistan and elsewhere [49]. This study did not directly examine the association between spousal communication about birth spacing and contraceptive use. However, it is likely that the strong association between contraceptive use and discussion of birth spacing with a relative or friend is largely driven by reports of women who received information about family planning or discussed birth spacing intentions with their husbands.

Furthermore, the fact that an individual woman’s odds of contraceptive use increase with the proportion of her neighbors who report having discussed birth spacing in the last year highlights the influence of one’s social environment on health behaviors [50]. This is a relatively conservative measure of openness to family planning, as it does not account for the role of the individual who discussed birth spacing with the woman, the nature of the conversation, or the accuracy of the information received. It does, however, suggest that there are benefits to continued investment in counseling, communication, and outreach to improve knowledge and increase discussion regarding contraceptive use. That the effect of receiving information from a Lady Health Worker loses statistical significance after adjusting for discussion of birth spacing in the community is consistent with studies in other locations where use of modern contraception appeared to be correlated with having discussed contraception with a community health worker but ceased to be statistically significant when a community-level discussion variable was introduced [51]. This provides insight into ways in which Lady Health Workers can increase family planning uptake, and suggests that it may beneficial to place greater focus on their work conducting support groups and community meetings where women and men gather to discuss matters pertinent to mother and child health, especially family planning. Finally, when controlling for other individual and community-level factors, the likelihood of a woman using contraception was reduced if she lived in a poorer community. This finding is consistent with studies in other contexts that show that residing in a wealthier community is associated with better health outcomes than residing in a community with a high concentration of extreme poverty [52].

Collectively, these findings highlight that programs need to adopt a multilevel approach to promoting contraceptive uptake, including investing in improving the quality of health services and shifting social norms to create an enabling environment for discussions about sexual and reproductive health choices. Understanding context-specific constraints and opportunities, however, is critical for scaling up family planning to reach national and international population and health development goals [53]. For example, although there is an essential health service package in place that has clearly defined guidelines and protocols for reproductive and maternal health services, provision of quality health services remains a challenge in many areas. There are districts in Sindh that do not have active health care providers, due to low levels of public financing for salaries and financial incentives in the private sector that lead to employment gaps and absenteeism [54]. A community midwife cadre has been introduced to fill some of this gap, but many midwives struggle to provide effective maternal health services in rural areas because they lack sufficient resources to deliver services in their catchment areas and leadership support to facilitate integration in the district health system [55]. Overcoming these gaps will require improvements in health system management as well as institutionalization of quality improvement initiatives to strengthen maternal and reproductive health services at the district, facility, and community levels [56]. Similarly, if Lady Health Workers are encouraged to focus more on facilitating interpersonal and community dialogue to reduce stigma around family planning additional investment will be needed to address issues such as payment delays, job insecurity, and being overworked because of conflicting priorities [57–59].

### Strengths and limitations

To the best of our knowledge, no previous study has examined the relationship between discussions of birth spacing and contraceptive use among postpartum women, using multilevel analysis to disentangle individual/household and community-level effects of health service provision on contraceptive use in Pakistan. This work has practical implications for health program implementation in Pakistan, and it provides a rich example of how contraceptive use is influenced by multiple socioecological factors. The study also has limitations, some of which are common to all observational studies that make inferences based on correlations from cross-sectional data, and others of which are unique to this study. First, because the data is cross-sectional, causal inferences about the effects of explanatory variables on contraceptive use are not possible. For example, it is assumed that discussing birth spacing leads to contraceptive use, but the reverse may also be true—that is, using contraception may have prompted a discussion of birth spacing with a health worker or family member. Second, community-level variables were based on the aggregation of individual-level data. A stronger design would link independent community-level data with individual-level data and would examine cross-level interaction effects. Third, there were no provisions in the dataset to examine whether postpartum family planning counseling was provided as part of antenatal or postpartum care or to ascertain the context, content, or depth of discussions women had about birth spacing. Information on both of these topics would have enriched the analysis and allowed for more precise interpretation of effects.

## Conclusions

This study showed community characteristics to have independent effects on individual propensity to use contraception among women within two years postpartum in Sindh, Pakistan. Increased coverage of quality antenatal care, increased reach of information about birth spacing, and living in a community with a high concentration of poverty were found to increase the odds of contraceptive use. Collectively, these findings highlight the need for programs to adopt a multilevel approach to promoting contraceptive uptake, including investing in efforts to improve counseling on healthy timing and spacing of pregnancies during antenatal care and community engagement to create an enabling environment for women and families to birth spacing and make informed choices about family planning.

## References

[1] Cleland J, Bernstein S, Ezeh A, Faundes A, Glasier A, Innis J. Family planning: the unfinished agenda. Lancet. 2006;368(9549):1810–27. 10.1016/S0140-6736(06)69480-4 . 17113431

[2] Ahmed S, Li Q, Liu L, Tsui AO. Maternal deaths averted by contraceptive use: an analysis of 172 countries. Lancet. 2012;380(9837):111–25. 10.1016/s0140-6736(12)60478-4 22784531

[3] Ezeh AC, Bongaarts J, Mberu B. Global population trends and policy options. Lancet. 2012;380c(9837):142–8. 10.1016/S0140-6736(12)60696-5 . 22784532

[4] Goldie SJ, Sweet S, Carvalho N, Natchu UC, Hu D. Alternative strategies to reduce maternal mortality in India: a cost-effectiveness analysis. PLoS Med. 2010;7(4):e1000264 10.1371/journal.pmed.1000264 20421922 PMC2857650

[5] Bongaarts J, Sathar Z, Mahmood A. Capturing the Demographic Dividend in Pakistan, Chapter Two: Population Trends in Pakistan. Population Council Book Series. 2013;1(1):10.

[6] National Institute of Population Studies (NIPS), ICF International. Pakistan Demographic and Health Survey 2012–13. Islamabad, Pakistan and Calverton, Maryland, USA: NIPS and ICF International, 2013.

[7] World Health Organization (WHO), United States Agency for International Development (USAID), Maternal and Child Health Integrated Program (MCHIP). Programming strategies for postpartum family planning. World Health Organization, 2013.

[8] Government of Pakistan. Pakistan Economic Survey, 2012–2013. Islamabad, Pakistan: Finance Division, Economic Advisor's Wing, 2013.

[9] UKAID, Bill and Melinda Gates Foundation. London Summit on Family Planning: Summaries of Commitments. 2012.

[10] Sathar ZA. Family planning: a missing priority in Pakistan's health sector? The Lancet. 2013;381(9884):2140–1. 10.1016/s0140-6736(13)60763-1 23684258

[11] Sultan M, Cleland JG, Ali MM. Assessment of a new approach to family planning services in rural Pakistan. American journal of public health. 2002;92(7):1168–72. 12084703 10.2105/ajph.92.7.1168 PMC1447209

[12] Douthwaite M, Ward P. Increasing contraceptive use in rural Pakistan: an evaluation of the Lady Health Worker Programme. Health Policy Plan. 2005;20(2):117–23. 10.1093/heapol/czi014 . 15746220

[13] Hardee K, Leahy E. Population, Fertility and Family Planning in Pakistan: A Program in Stagnation. 2008;3(3).

[14] Mumtaz Z, Salway S, Nykiforuk C, Bhatti A, Ataullahjan A, Ayyalasomayajula B. The role of social geography on Lady Health Workers' mobility and effectiveness in Pakistan. Soc Sci Med. 2013;91:48–57. 10.1016/j.socscimed.2013.05.007 . 23849238

[15] Abbas K. Costs and utilization of public sector family planning services in Pakistan. Journal of the Pakistan Medical Association. 2012. 24386728

[16] Carton TW, Agha S. Changes in contraceptive use and method mix in Pakistan: 1990–91 to 2006–07. Health Policy Plan. 2012;27(2):166–74. 10.1093/heapol/czr022 . 21441567

[17] Shah NM, Wang W, Bishai DM. Comparing private sector family planning services to government and NGO services in Ethiopia and Pakistan: how do social franchises compare across quality, equity and cost? Health Policy Plan. 2011;26 Suppl 1:i63–71. 10.1093/heapol/czr027 21729919 PMC3606031

[18] Pakistan Bureau of Statistics. Contraceptive Performance Report, 2013–2014. Islamabad, Paksitan: Government of Pakistan Statistics Division, 2014.

[19] Nishtar NA, Sami N, Alim S, Pradhan N, Hasnain FU. Determinants of contraceptives use amongst youth: an exploratory study with family planning service providers in Karachi Pakistan. Global journal of health science. 2013;5(3):1–8. 10.5539/gjhs.v5n3p1 . 23618469 PMC4776775

[20] Bruce J. Fundamental elements of the quality of care: a simple framework. Stud Fam Plann. 1990;21(2):61–91. . 2191476

[21] Casterline J, Sathar Z, ul Haque M. Obstacles to Contraceptive Use in Pakistan: A Study in Punjab. Studies in Family Planning. 2001;32(2):15. 10.1111/j.1728-4465.2001.00095.x 11449867

[22] Agha S. Intentions to use contraceptives in Pakistan: implications for behavior change campaigns. BMC Public Health. 2010;10:450 10.1186/1471-2458-10-450 20673374 PMC2920282

[23] Hameed W, Azmat SK, Ali M, Sheikh MI, Abbas G, Temmerman M, et al Women's empowerment and contraceptive use: the role of independent versus couples' decision-making, from a lower middle income country perspective. PLoS One. 2014;9(8):e104633 10.1371/journal.pone.0104633 25119727 PMC4131908

[24] Bernard P, Charafeddine R, Frohlich KL, Daniel M, Kestens Y, Potvin L. Health inequalities and place: a theoretical conception of neighbourhood. Soc Sci Med. 2007;65(9):1839–52. Epub 2007/07/07. 10.1016/j.socscimed.2007.05.037 . 17614174

[25] Nishtar NA, Sami N, Faruqi A, Khowaja S, Ul-Hasnain F. Myths and fallacies about male contraceptive methods: a qualitative study amongst married youth in slums of Karachi, Pakistan. Global journal of health science. 2013;5(2):84–93. 10.5539/gjhs.v5n2p84 . 23445697 PMC4776822

[26] Marvi K, Howard N. Objects of temporary contraception: an exploratory study of women's perspectives in Karachi, Pakistan. BMJ open. 2013;3(8). 10.1136/bmjopen-2013-003279 23906959 PMC3733316

[27] Mahmood N. Reproductive goals and family planning attitudes in Pakistan: a couple-level analysis. Pakistan development review. 1998;37(1):19–34. Epub 2002/09/28. . 12349418

[28] Azmat SK, Ahmed S, Hameed W, Bilgrami M, Khan A, Khan AA, et al Performance and measurement of a community-based distribution model of family planning services in Pakistan. JPMA The Journal of the Pakistan Medical Association. 2013;63(4 Suppl 3):S40–5. . 24386729

[29] Elfstrom KM, Stephenson R. The role of place in shaping contraceptive use among women in Africa. PLoS One. 2012;7(7):e40670 Epub 2012/07/21. 10.1371/journal.pone.0040670 22815784 PMC3399881

[30] Steele F, Curtis SL, Choe M. The impact of family planning service provision on contraceptive-use dynamics in Morocco. Studies in family planning. 1999;30(1):28–42. . 10216894 10.1111/j.1728-4465.1999.00028.x

[31] Bongaarts J, Bruce J. The causes of unmet need for contraception and the social content of services. Studies in family planning. 1995;26(2):57–75. . 7618196

[32] Few R, Harpham T, Atkinson S. Urban primary health care in Africa: a comparative analysis of city-wide public sector projects in Lusaka and Dar es Salaam. Health & place. 2003;9(1):45–53. 10.1016/s1353-8292(02)00029-1 . 12609472

[33] Stephenson R, Baschieri A, Clements S, Hennink M, Madise N. Contextual influences on modern contraceptive use in sub-Saharan Africa. American journal of public health. 2007;97(7):1233–40. 10.2105/AJPH.2005.071522 17538071 PMC1913073

[34] Kyegombe N, Abramsky T, Devries KM, Starmann E, Michau L, Nakuti J, et al The impact of SASA!, a community mobilization intervention, on reported HIV-related risk behaviours and relationship dynamics in Kampala, Uganda. Journal of the International AIDS Society. 2014;17:19232 10.7448/IAS.17.1.19232 25377588 PMC4223282

[35] Jacobstein R, Curtis C, Spieler J, Radloff S. Meeting the need for modern contraception: effective solutions to a pressing global challenge. Int J Gynaecol Obstet. 2013;121 Suppl 1:S9–15. Epub 2013/03/14. 10.1016/j.ijgo.2013.02.005 . 23481357

[36] Kish L. A Procedure for Objective Respondent Selection within the Household. Journal of the American Statistical Association. 1949;44(247):7.

[37] Davis R, Luna J, Rodriguez-Lainz A, Sarriot E. The Rapid Household Survey: How to Obtain Reliable Data on Health at the Local Level. Calverton, MD: ICF Macro, 2009.

[38] Benefo KD. The determinants of the duration of postpartum sexual abstinence in West Africa: a multilevel analysis. Demography. 1995;32(2):139–57. Epub 1995/05/01. . 7664957

[39] Gupta N. Sexual initiation and contraceptive use among adolescent women in northeast Brazil. Studies in family planning. 2000;31(3):228–38. Epub 2000/10/06. . 11020934 10.1111/j.1728-4465.2000.00228.x

[40] Hamid S, Stephenson R. Provider and health facility influences on contraceptive adoption in urban Pakistan. International family planning perspectives. 2006;32(2):71–8. Epub 2006/07/14. 10.1363/ifpp.32.071.06 . 16837387

[41] Dehlendorf C, Levy K, Ruskin R, Steinauer J. Health care providers' knowledge about contraceptive evidence: a barrier to quality family planning care? Contraception. 2010;81(4):292–8. 10.1016/j.contraception.2009.11.006 20227544 PMC2892417

[42] Hodgins S, D'Agostino A. The quality-coverage gap in antenatal care: toward better measurement of effective coverage. Global Health: Science and Practice. 2014;2(2):173–81. 10.9745/ghsp-d-13-00176 PMC4168625 25276575

[43] Andrew EV, Pell C, Angwin A, Auwun A, Daniels J, Mueller I, et al Factors affecting attendance at and timing of formal antenatal care: results from a qualitative study in Madang, Papua New Guinea. PLoS One. 2014;9(5):e93025 10.1371/journal.pone.0093025 24842484 PMC4026245

[44] Gross K, Alba S, Glass TR, Schellenberg JA, Obrist B. Timing of antenatal care for adolescent and adult pregnant women in south-eastern Tanzania. BMC Pregnancy Childbirth. 2012;12:16 10.1186/1471-2393-12-16 22436344 PMC3384460

[45] Kyei NN, Campbell OM, Gabrysch S. The influence of distance and level of service provision on antenatal care use in rural Zambia. PLoS One. 2012;7(10):e46475 10.1371/journal.pone.0046475 23056319 PMC3464293

[46] Ong L. Doctor-patient communication: a review of the literature. BMC Public Health. 1995. 10.1016/0277-9536(94)00155-m 7792630

[47] Goodyear-Smith F, Buetow S. Power Issues in the Doctor-Patient Relationship. Health Care Analysis. 2001. 10.1023/A:1013812802937 11874258

[48] Moazam F. Families, Patients, and Physicians in Medical Decisionmaking: A Pakistani Perspective. The Hastings Center Report. 2000;30(6):28–37. 10.2307/3528451 11475993

[49] Kamran I, Arif MS, Vassos K. Concordance and discordance of couples living in a rural Pakistani village: perspectives on contraception and abortion—a qualitative study. Global public health. 2011;6 Suppl 1:S38–51. Epub 2011/07/08. 10.1080/17441692.2011.590814 . 21732869

[50] Dynes M, Stephenson R, Rubardt M, Bartel D. The influence of perceptions of community norms on current contraceptive use among men and women in Ethiopia and Kenya. Health & place. 2012;18(4):766–73. Epub 2012/05/15. 10.1016/j.healthplace.2012.04.006 . 22579117

[51] Kayembe PK, Fatuma AB, Mapatano MA, Mambu T. Prevalence and determinants of the use of modern contraceptive methods in Kinshasa, Democratic Republic of Congo. Contraception. 2006;74(5):400–6. 10.1016/j.contraception.2006.06.006 . 17046382

[52] Kayode GA, Amoakoh-Coleman M, Agyepong IA, Ansah E, Grobbee DE, Klipstein-Grobusch K. Contextual risk factors for low birth weight: a multilevel analysis. PLoS One. 2014;9(10):e109333 10.1371/journal.pone.0109333 25360709 PMC4215836

[53] Byrne A, Morgan A, Soto EJ, Dettrick Z. Context-specific, evidence-based planning for scale-up of family planning services to increase progress to MDG 5: health systems research. Reproductive health. 2012;9(27):27 10.1186/1742-4755-9-27 23140196 PMC3563623

[54] Nishtar S. The mixed health systems syndrome. Bull World Health Organ. 2010;88(1):74–5. Epub 2010/04/30. 10.2471/BLT.09.067868 20428356 PMC2802440

[55] Sarfraz M, Hamid S. Challenges in delivery of skilled maternal care—experiences of community midwives in Pakistan. BMC Pregnancy and Childbirth. 2014;14(1):59 10.1186/1471-2393-14-59 24499344 PMC3922011

[56] Nishtar S. The Gateway Paper—preventive and promotive programs in Pakistan and health reforms in Pakistan. JPMA The Journal of the Pakistan Medical Association. 2006;56(12 Suppl 4):S51–65. Epub 2007/06/29. . 17595833

[57] Hafeez A, Mohamud BK, Shiekh MR, Shah SA, Jooma R. Lady health workers programme in Pakistan: challenges, achievements and the way forward. JPMA The Journal of the Pakistan Medical Association. 2011;61(3):210–5. . 21465929

[58] Wazir MS, Shaikh BT, Ahmed A. National program for family planning and primary health care Pakistan: a SWOT analysis. Reproductive health. 2013;10(1):60 Epub 2013/11/26. 10.1186/1742-4755-10-60 24268037 PMC3842797

[59] Haq Z, Iqbal Z, Rahman A. Job stress among community health workers: a multi-method study from Pakistan. International journal of mental health systems. 2008;2(1):15 Epub 2008/10/29. 10.1186/1752-4458-2-15 18954470 PMC2584063

